# A186 INFLAMMATION MODIFIES DOSE-DEPENDENT RESPONSES OF INTESTINAL ANTI-TUMOUR MICRORNAS TO CRANBERRY PROANTHOCYANIDIN AND ITS MICROBIAL METABOLITE 3-(4-HYDROXYPHENYL)-PROPIONIC ACID

**DOI:** 10.1093/jcag/gwad061.186

**Published:** 2024-02-14

**Authors:** Z Dimoff, Z Lofft, F Liang, S Chen, I Paetau-Robinson, C Khoo, A Taibi, E Comelli

**Affiliations:** University of Toronto Temerty Faculty of Medicine, Toronto, ON, Canada; University of Toronto Temerty Faculty of Medicine, Toronto, ON, Canada; University of Toronto Temerty Faculty of Medicine, Toronto, ON, Canada; University of Toronto Temerty Faculty of Medicine, Toronto, ON, Canada; Ocean Spray Cranberries Inc, Middleboro, MA; Ocean Spray Cranberries Inc, Middleboro, MA; University of Toronto Temerty Faculty of Medicine, Toronto, ON, Canada; University of Toronto Temerty Faculty of Medicine, Toronto, ON, Canada

## Abstract

**Background:**

Cranberries are a rich source of proanthocyanidins (PAC), a type of polyphenol with anti-cancer properties. We previously found that PAC, along with its microbially-derived metabolite 3-(4-hydroxyphenyl)-propionic acid (HPPA), trigger unique regulatory responses of microRNAs (miRNAs) in intestinal epithelial cells including the upregulation of miR-146a-5p. Both miR-146a-5p and miR363-3p are anti-inflammatory and downregulate anti-tumorigenic signalling pathways such as the interleukin (IL)-17 and wingless-related integration site (Wnt) respectively. miR-146a-5p also attenuates IL-17-promoting cytokines levels (e.g., IL-6).

**Aims:**

To determine if miR-146a-5p and miR-363-3p respond to different physiologically relevant concentrations of PAC and HPPA in inflammatory and non-inflammatory conditions.

**Methods:**

Fully differentiated Caco-2BBe1 colonic epithelial cells were treated with two doses of PAC-enriched cranberry extract (50μg/ml, 100μg/ml), HPPA (5μg/ml, 10μg/ml), or with Dulbecco’s Modified Eagle Medium (DMEM; control) for 24 hours, followed by IL-1β (1ng/ml) or mock stimulation for three hours. Human IL-6 homogeneous time-resolved fluorescence (HTRF) kits were used to quantify IL-6 in cell supernatant. RNA was extracted and used for miRNA profiling using Nanostring technology. Statistical analysis was performed in R version 4.2.1 with the R-packages NanostringDiff, NanostringNorm, and Pheatmap.

**Results:**

At homeostasis, 42 and 2 (miR-146a and miR363-3p) miRNAs, respectively, uniquely responded to increasing PAC or HPPA concentrations. In the inflammatory state, no miRNAs responded to increasing concentrations of PAC and HPPA. However, the expression of miR-363-3p increased (qampersand:003C0.001) in response to 50μg/ml of PAC + IL-1β but decreased in response to 5μg/ml of HPPA + IL-1β , and the expression of miR-146a-5p increased (qampersand:003C0.001) in response to 5μg/ml of HPPA + IL-1β, and 50μg/ml PAC + IL-1β. Predicted miRNA gene-pathway analysis revealed that miR-146a-5p, miR-363-3p, and the other 42 miRNAs commonly target pathways involved in the tumorigenesis of colorectal cancer such as mitogen-activated protein kinases (MAPK), hedgehog, and Wnt pathways. Though, in inflamed cells, only HPPA (5 or 10 μg/ml) attenuated IL-6 secretion (pampersand:003C0.05), which may be driven by increased expression of miR-146a-5p.

**Conclusions:**

These findings suggest that cranberries proanthocyanidins and their metabolites affect miRNAs involved in cancer related pathways in different manners and concentrations, which may depend on the inflammatory status. The gut microbiota may be partially responsible for unlocking these effects.

**Funding Agencies:**

Ocean Spray Cranberries, Inc. and the Natural Sciences of Engineering Research Council of Canada (NSERC)

